# Identification of a Novel Enhancer/Chromatin Opening Element Associated with High-Level γ-Globin Gene Expression

**DOI:** 10.1128/MCB.00197-18

**Published:** 2018-09-14

**Authors:** Yong Shen, MacLean A. Bassett, Aishwarya Gurumurthy, Rukiye Nar, Isaac J. Knudson, Cameron R. Guy, Alex Perez, Russell W. Mellen, Masatoshi Ikeda, Mir A. Hossain, Suming Huang, Kazuhiko Igarashi, Jörg Bungert

**Affiliations:** a Department of Biochemistry and Molecular Biology, College of Medicine, Health Cancer Center, Genetics Institute, Center for Epigenetics, University of Florida, Gainesville, Florida, USA; b Department of Biochemistry, Tohoku University Graduate School of Medicine, Sendai, Miyagi, Japan

**Keywords:** globin, enhancer, histones, zinc finger DNA-binding domain, transcription, chromatin, gene regulation, hemoglobin, synthetic zinc-finger DNA-binding domain, zinc finger proteins

## Abstract

The organization of the five β-type globin genes on chromosome 11 reflects the timing of expression during erythroid cell development, with the embryonic ε-globin gene being located at the 5′ end, followed by the two fetal γ-globin genes, and with the adult β- and δ-globin genes being located at the 3′ end. Here, we functionally characterized a DNase I-hypersensitive site (HS) located 4 kb upstream of the Gγ-globin gene (HBG-4kb HS). This site is occupied by transcription factors USF1, USF2, EGR1, MafK, and NF-E2 in the human erythroleukemia cell line K562 and exhibits histone modifications typical for enhancers. We generated a synthetic zinc finger (ZF) DNA-binding domain targeting the HBG-4kb HS (HBG-4kb ZF). The HBG-4kb ZF interacted with the target site *in vitro* and in the context of cells with a high affinity and specificity. Direct delivery of the HBG-4kb ZF to K562 and primary human erythroid cells caused a reduction in γ-globin gene expression which was associated with decreased binding of transcription factors and active histone marks at and downstream of the HS. The data demonstrate that the HBG-4kb HS is important for fetal globin production and suggest that it may act by opening chromatin in a directional manner.

## Abstract (precis)

The organization of the five β-type globin genes on chromosome 11 reflects the timing of expression during erythroid cell development, with the embryonic ε-globin gene being located at the 5′ end, followed by the two fetal γ-globin genes, and with the adult β- and δ-globin genes being located at the 3′ end. Here, we functionally characterized a DNase I-hypersensitive site (HS) located 4 kb upstream of the Gγ-globin gene (HBG-4kb HS).

## INTRODUCTION

Enhancers are DNA elements that activate transcription of genes in a position- and orientation-independent manner (1). Active or poised enhancers establish DNase I hypersensitive sites (HSs), which are usually 200 to 400 bp long and which are characterized by a nucleosome-free region or a segment of DNA at which the nucleosome structure is altered. Enhancers are bound by ubiquitous and/or tissue-restricted transcription factors and coregulatory protein complexes. Nucleosomes flanking enhancers contain H3K4 monomethylation (H3K4me1) and H3K27 acetylation (H3K27ac) marks (2). Furthermore, a large fraction of enhancers initiate transcription to produce enhancer RNA (eRNA) (3). Some genes or gene loci are regulated by multiple enhancers that operate together in the context of locus control regions (LCRs) or superenhancers (4–7). There is no unifying model for enhancer function. Rather, enhancers act in different ways to stimulate target gene expression, including opening of the chromatin structure, relocation of genes to active sites in the nucleus, transfer of components of the transcription complex to the promoter, and/or stimulation of the transition from paused to elongation-active transcription complexes (8–10).

The organization of the β-type globin genes reflects the order by which they are expressed during development, with the embryonic ε-globin gene being located at the 5′ end and the adult genes being located at the 3′ end. High-level expression of the genes is regulated by the LCR (11). The β-globin LCR is composed of multiple HS elements that function together to mediate high-level globin gene expression (4, 5). The LCR HS elements function synergistically at ectopic sites and additively at the endogenous mouse globin gene locus (12, 13). Together they mediate extremely high levels of globin gene expression, and the LCR is thus similar in function to superenhancers. The stage-specific expression of the β-type globin genes is regulated by gene proximal promoter and enhancer elements (14). These DNA elements associate with transcription factors that activate or repress transcription of the genes at specific developmental stages.

Correct expression of the two fetal γ-globin genes (the Gγ- and Aγ-globin genes) is regulated by positive and negative *cis*-regulatory DNA elements that are distal to the genes, e.g., 3′ enhancers, or located in the proximal promoter regions (15). The hereditary persistence of fetal hemoglobin (HPFH) is characterized by a failure to efficiently suppress γ-globin expression in the adult (15). Several large deletions encompassing the adult β-type globin genes are associated with HPFH. These deletions bring enhancer elements in close proximity to the γ-globin genes. Other mutations causing HPFH are located in the γ-globin promoter regions, in which they alter the binding of repressor proteins or create binding sites for transcriptional activators (15). Furthermore, mutations in DNA sequences regulating expression of the transcription repressor B cell chronic lymphocytic leukemia (CLL)/lymphoma 11A (BCL11A) have been linked to elevated expression of fetal hemoglobin (16, 17). Recent studies have identified novel *cis*-regulatory DNA elements (e.g., HBD-1kb and BGL3-HBBP1) across the human β-globin gene locus that play significant roles in the regulation of γ-globin gene expression (18–20). The significance of studying the regulation of the γ-globin genes is based on the fact that continued expression of γ-globin in the adult prevents severe phenotypes associated with sickle cell disease (SCD) or β-thalassemias (21).

Here we interrogated the function of a hitherto uncharacterized DNase I HS located 4 kb upstream of the Gγ-globin gene (HBG-4kb HS). Data from the Encyclopedia of DNA Elements (ENCODE) show that the HBG-4kb HS is associated with histone marks typically found at enhancers (22, 23). In addition, this site is bound to ubiquitously expressed and tissue-restricted transcription factors, including EGR1, USF1, USF2, MafK, and NF-E2. We generated a synthetic zinc finger (ZF) DNA-binding domain (DBD) targeting this site (HBG-4kb ZF) and delivered it to K562 and primary erythroid cells. Exposure of these cells to the HBG-4kb ZF led to a reduction of enhancer- and promoter-associated histone modifications and a decrease in γ-globin gene expression. We also observed a decrease in γ-globin expression in K562 cells harboring CRISPR/Cas9-mediated deletions within the HBG-4kb HS.

## RESULTS

**FIG 1:**
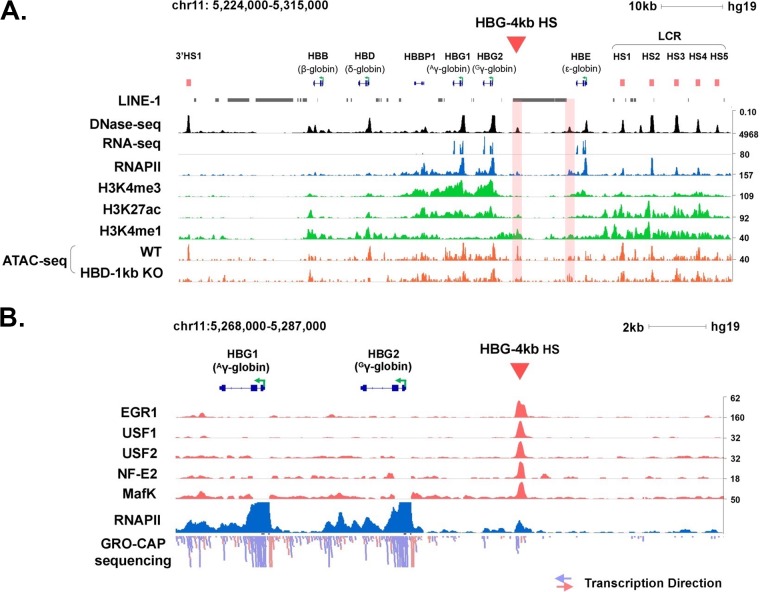
The HBG-4kb HS is characterized by elevated levels of H3K4me1 and H3K27ac and is bound by transcription factors and Pol II in K562 cells. (A) Data from the ENCODE project showing the levels of H3K4me1, H3K4me3, H3K27ac, Pol II (RNAPII), and RNA (RNA-seq) in the human β-globin gene locus in K562 cells. ATAC-seq data from wild-type K562 cells and K562 cells lacking the upstream δ-globin HS at kb −1 (HBD-1kb) are shown at the bottom. (B) Interaction of transcription factors EGR1, USF1, USF2, NFE2, and MafK as well as Pol II (RNAPII) with the HBG-4kb HS. Also shown are GRO-cap sequencing data revealing low-level transcription toward the γ-globin genes. Data were retrieved from the ENCODE project and GEO database. The triangles point to the HBG-4kb HS. The numbers on the right represent reads per kilobase per million mapped reads (RPKM).

The analysis of publicly available data from the ENCODE project revealed the presence of a DNase I HS located about 4 kb upstream of the Gγ-globin transcription start site (HBG-4kb HS) (red arrowhead in Fig. 1A) in K562 cells (22, 23). This site is associated with elevated levels of H3K27 acetylation (H3K27ac) and H3K4 monomethylation (H3K4me1), while the γ-promoter regions are associated with elevated levels of H3K4 trimethylation (H3K4me3). H3K27ac and H3K4me1 marks are typically associated with active enhancer elements. Interestingly, none of these active marks are found immediately upstream of the HBG-4kb HS, and the H3K4me1 mark extends unidirectionally toward the Gγ-globin promoter. Previous studies have shown that deletion of a DNase I HS 1 kb upstream of the adult δ-globin gene (HBD-1kb HS) reduced chromatin accessibility, as determined by assay for transposase-accessible chromatin using sequencing (ATAC-seq), at a distal HS (3′ HS1) and at the Gγ-globin HS at kb −4 (Fig. 1A) (19) by more than 10-fold (see Table S6 in the supplemental material).

The HBG-4kb HS element appears to be a hot spot for transcription factor binding in the human erythroleukemia cell line K562, which expresses high levels of γ-globin. Inspecting chromatin immunoprecipitation sequencing (ChIP-seq) data from the ENCODE project, we found that the HBG-4kb HS interacts with a large number of transcription factors that are either ubiquitously expressed or more restricted to hematopoietic tissues. Among the factors interacting with this element are EGR1, USF1, USF2, NF-E2, and MafK (Fig. 1B). The association of USF and NF-E2 transcription factors is interesting, as we have shown before that they interact with each other and are implicated in the recruitment of transcription complexes to the β-globin gene locus (24). Indeed, RNA polymerase II (Pol II) is associated with the HBG-4kb HS, and global nuclear run-on of 5′-capped RNA (GRO-cap) sequencing data show that transcription initiates within this region and proceeds primarily in a unidirectional manner toward the Gγ-globin gene. Upstream of the HBG-4kb HS is a long interspersed element 1 (LINE-1) that is conserved among primate species (Fig. S1).

**FIG 2:**
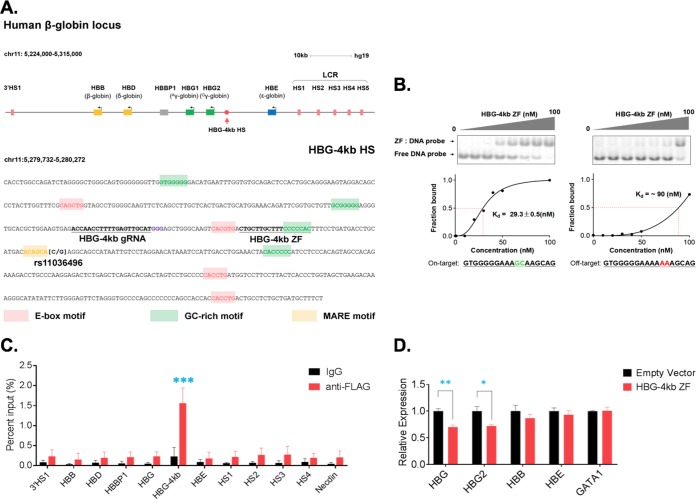
Generation and assessment of DNA-binding specificity and affinity of a ZF-DBD targeted to the HBG-4kb HS. (A) Outline of the human β-globin gene locus and the sequence of the HBG-4kb HS. DNA sequences highlighted in green, red, and yellow represent potential transcription factor binding sites, as indicated. Underlined sequences were targeted by a guide RNA for CRISPR/Cas9-mediated deletions or targeted by the HBG-4kb ZF. The HBG-4kb ZF contained 6 zinc finger modules and was targeted to an 18-bp sequence overlapping the EGR1 transcription factor binding site. The C/G SNP (rs11036496) represents a transversion mutation associated with hyperuricemia. (B) Assessment of the DNA-binding affinity and specificity of the HBG-4kb ZF *in vitro* by EMSAs. Fluorescently labeled double-stranded DNA oligonucleotides containing the HBG-4kb ZF target site (left) or a mutant DNA sequence (right) were incubated without or with increasing concentrations of the HBG-4kb ZF, as indicated. After gel electrophoresis, the intensity of the free DNA and the DNA in the context of the protein/DNA complex was measured and a binding curve plotting the fraction of DNA bound against the protein concentration was generated. The *K_d_* reflects the concentration of the protein at which half of the DNA was bound. (C) ChIP assay examining the binding of the HBG-4kb ZF to specific sites in the β-globin gene locus as well as to the necdin promoter, as indicated. K562 cells were transfected with a viral vector expressing the FLAG-tagged HBG-4kb ZF and subjected to ChIP using antibodies specific for the FLAG tag or a negative-control IgG antibody, as indicated. The error bars reflect standard errors of the means (SEM) from three independent biological replicates. (D) RT-qPCR analysis of total γ-globin (HBG), Gγ-globin (HBG2), β-globin (HBB), ε-globin (HBE), and GATA1 gene expression in K562 cells expressing the HBG-4Kb ZF or in cells harboring the empty vector. The error bars reflect the SEM from three independent experiments (***, *P* < 0.001; **, *P* < 0.01; *, *P* < 0.05).

Curiously, within the HBG-4kb HS there is a single nucleotide polymorphism (SNP) (rs11036496) that is associated with hyperuricemia in African Americans (Fig. 2A) (25). This SNP is characterized by a C-to-G transversion located close to a Maf recognition element (MARE) and downstream of the EGR1 transcription factor binding site. To examine the function of the HBG-4kb HS, we generated a synthetic ZF-DBD targeting this site (HBG-4kb ZF) (Fig. 2A). We previously demonstrated that ZF-DBDs without effector domains are efficient in competing for endogenous transcription factors at specific sites (26–28). Furthermore, these proteins can be directly delivered to mammalian cells and locate to the nucleus even in the absence of a nuclear localization signal (29, 30). The HBG-4kb ZF contained 6 ZFs and a His tag which was used to purify it from Escherichia coli. We first determined the DNA-binding affinity and specificity *in vitro* using electrophoretic mobility shift assays (EMSAs). We incubated increasing concentrations of purified recombinant HBG-4kb ZF with a constant amount of fluorescently labeled double-stranded DNA oligonucleotides and resolved the free DNA and the protein-DNA complexes on native polyacrylamide gels (Fig. 2B). The dissociation constant (*K_d_*) reflects the concentration of protein at which half of the DNA molecules are bound and was determined to be 29.5 nM for the HBG-4kb ZF. Changing only 2 nucleotides in the target sequence (indicated in green and red in Fig. 2A) reduced the DNA-binding affinity 3-fold (from 29.5 to 90 nM). Furthermore, binding to the wild-type DNA sequence revealed a sigmoid binding curve suggesting cooperativity among the zinc fingers. To examine the DNA-binding specificity of the HBG-4kb ZF in the context of cells, we transfected K562 cells with a viral construct expressing the FLAG-tagged HBG-4kb ZF to generate a stable cell line. The transfected cells were subjected to chromatin immunoprecipitation (ChIP) (Fig. 2C). The precipitation with FLAG-specific antibodies but not with control IgG antibodies enriched for the HBG-4kb HS (about 6-fold). The binding of the HBG-4kb ZF to the HBG-4kb HS was highly specific, as we did not observe elevated levels of enrichment at LCR HSs, the globin gene promoters, 3′HS1, HBBP1, or the necdin gene promoter. Transfected K562 cells expressing the HBG-4kb ZF revealed decreased Gγ-globin (HBG2) and total γ-globin (HBG) expression but no change in expression of embryonic ε-globin (HBE), adult β-globin (HBB), or GATA1 (Fig. 2D). Expression of these genes was not affected in K562 cells transfected with the empty vector.

**FIG 3:**
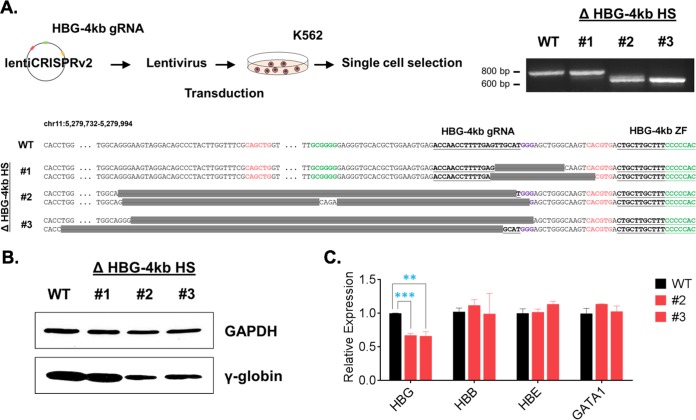
Reduced γ-globin gene expression in K562 cells harboring deletions within the HGB-4kb HS. (A) (Top left) K562 cells were transduced with lentivirus expressing Cas9 and a HGB-4kb specific guide RNA (gRNA). (Top right) Three single-cell clones (labeled #1, #2, and #3) were selected and subjected to PCR using primers flanking the HBG-4kb HS. (Bottom) The DNA sequences of the PCR products are shown. E boxes that interact with USF are highlighted in red, and GC-rich binding sites are highlighted in green. Gray bars delineate the extent of the deletions in the two alleles of the three clones. The underlined bold sequences with the adjacent purple sequences represent the target for the guide RNA and PAM sequence. The bold underlined sequence at the right represents the binding site for the HBG-4kb ZF. Dotted regions represent gaps in the sequences not shown. (B) Western blotting of GAPDH and γ-globin expression in WT K562 cells as well as in the three single-cell clones carrying deletions within the HBG-4kb HS. (C) RT-qPCR expression analysis of γ-globin (HBG), β-globin (HBB), ε-globin (HBE), and GATA1 in WT cells and in K562 single-cell clones 2 and 3 harboring deletions within the HBG-4kb HS. Error bars reflect the SEM from three independent RNA isolations with qPCR performed in triplicate (***, *P* < 0.001; **, *P* < 0.01).

To obtain additional evidence that the HBG-4kb HS functions as an enhancer of γ-globin gene expression, we deleted sequences within the HBG-4kb using the CRISPR/Cas9 technology. We designed a specific guide RNA (gRNA) targeting the center of the HBG-4kb and used lentivirus transduction to deliver the guide RNA together with Cas9 to K562 cells, as outlined in Fig. 3A. We generated single-cell clones from cells expressing Cas9 and the guide RNA and from wild-type (WT) cells. We used PCR with primers spanning the HBG-4kb HS to identify clones with deletions in this region. We continued with three K562 cell clones that harbor various deletions within the HBG-4kb HS. Clone 1 contained 16- and 24-bp deletions overlapping and downstream of sequences targeted by the guide RNA. Clone 2 contained 147- and 149-bp deletions upstream and downstream of the target site. The allele with the 149-bp deletion contained a 4-bp insertion, as indicated in Fig. 3A. Clone 3 harbored 140- and 215-bp deletions upstream of the target site. The K562 cells were subjected to karyotype analysis, which revealed heterogeneity with respect to the copy number of chromosome 11 (chr 11). Most cells contained three copies of chromosome 11, with one copy often harboring a large deletion of the p-arm encompassing the β-globin gene locus. There is no evidence that the edited K562 cell clones contained a third copy of the β-globin gene locus. The cells were subjected to Western blotting, and cells from K562 clones 2 and 3 revealed a decrease in expression of the γ-globin protein compared to WT cells or clone 1 cells (Fig. 3B). RNA expression analysis confirmed that the larger deletions within the HBG-4kb HS reduced expression of the γ-globin gene but not that of the adult β-globin (HBB), embryonic ε-globin (HBE), or GATA1 genes (Fig. 3C). These data provide additional evidence demonstrating that the HBG-4kb HS functions as a positive regulator of fetal globin expression and validates the approach of using the HBG-4kb ZF to further functionally analyze this element.

**FIG 4:**
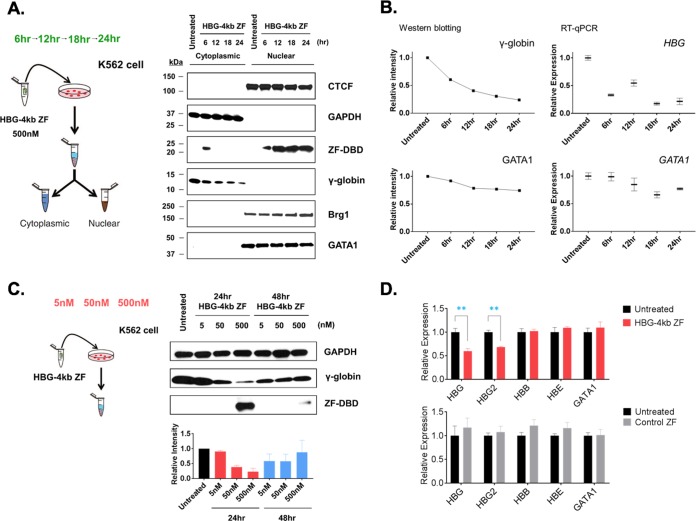
Reduced γ-globin expression in K562 cells exposed to the HBG-4kb ZF. (A) Temporal effect of the HBG-4kb ZF on gene expression in K562 cells. K562 cells were exposed to 500 nM HBG-4kb ZF for 6 to 24 h, as indicated. Untreated cells were also examined as indicated. Cells were lysed, the lysates were compartmentalized into nuclear and cytoplasmic fractions, and the proteins were subjected to Western blotting using antibodies specific for CTCF, GAPDH, HBG-4kb ZF (ZF-DBD), γ-globin, Brg1, and GATA1, as shown. (B) Effect of the HBG-4kb ZF on γ-globin and GATA1 RNA and protein levels during the course of exposure to the HBG-4kb ZF. Proteins and RNA were extracted and subjected to Western blotting (using γ-globin- and GATA1-specific antibodies) or RT-qPCR using primers specific for the γ-globin (HBG) or GATA1 genes. The RT-qPCR data were from three independent experiments, and the error bars represent the SEM. (C) Assessment of ZF-DBD dosage effect. K562 cells were exposed to different concentrations (5, 50, and 500 nM) of the HBG-4kb ZF for 24 or 48 h. After exposure to the ZF-DBD, the K562 cells were subjected to Western blotting experiments with antibodies specific for GAPDH, γ-globin, or the HBG-4kb ZF (αZF). The graph on the bottom reflects the quantitation of signals from 3 independent experiments, with the error bars representing the SEM. (D) Exposure of K562 cells to the HBG-4kb ZF specifically reduced γ-globin gene expression. K562 cells were exposed to 500 nM HBG-4kb ZF (top) or to 500 nM negative-control ZF-DBD (bottom) for 12 h. RNA was isolated, reverse transcribed, and subjected to qPCR using primers specific for the γ-globin (HBGs), Gγ-globin (HBG2), adult β-globin (HBB), embryonic ε-globin (HBE), and GATA1 genes, as indicated. Expression in K562 cells not exposed to the ZF-DBDs (Untreated) was set at 1. Error bars reflect the SEMs from three independent biological repeats (**, *P* < 0.01).

Next, we directly delivered the HBG-4kb ZF to K562 cells and obtained cytoplasmic and nuclear protein fractions at different time points after delivery of the protein. The fractions were then subjected to Western blotting using antibodies specific for CTCF, GAPDH (glyceraldehyde-3-phosphate dehydrogenase), HBG-4kb ZF (ZF-DBD, anti-ZF), γ-globin, Brg1, and GATA1 (Fig. 4A). The data show that the HBG-4kb ZF was first detectable in the cytoplasm 6 h after delivery and subsequently located exclusively to the nucleus. Expression of γ-globin decreased in a gradual manner beginning after 6 h and reached its lowest levels 24 h after delivery of the ZF-DBD. Expression of the other proteins located in the cytoplasm (GAPDH) or nucleus (CTCF, GATA1, and Brg1) was not affected by the presence of the HBG-4kb ZF. These data demonstrate that the HBG-4kb ZF specifically reduced expression of the γ-globin gene and suggest that the HBG-4kb HS functions as an enhancer. Comparison of the mRNA and protein levels for γ-globin and GATA1 demonstrates that the HBG-4kb ZF caused a rapid decrease in γ-globin gene transcription, which was followed by a decrease in protein levels, while GATA1 protein and mRNA levels did not change (Fig. 4B). We next examined γ-globin expression by Western blotting in K562 cells exposed to different concentrations of the HBG-4kb ZF (5, 50, and 500 nM) for 24 and 48 h (Fig. 4C). We observed a gradual decrease in γ-globin expression in a manner dependent on the dose of the HBG-4kb ZF. There was less of an effect on γ-globin after 48 h, which was due to the instability of the ZF-DBD, which was barely detectable after 48 h. Expression of GAPDH did not change in these experiments. Exposure of K562 cells to the HBG-4kb ZF specifically reduced the mRNA levels of the Gγ-globin and overall γ-globin gene levels without affecting expression of the adult β-globin, embryonic ε-globin, or GATA1 genes (Fig. 4D). We delivered a negative-control ZF-DBD (control ZF), which was designed to target the 158 region of the γ-globin promoter region, to K562 cells and examined gene expression (Fig. 4D) (31). The results show that the control ZF did not alter expression of the GATA1 or the ε-, γ-, and β-globin genes.

**FIG 5:**
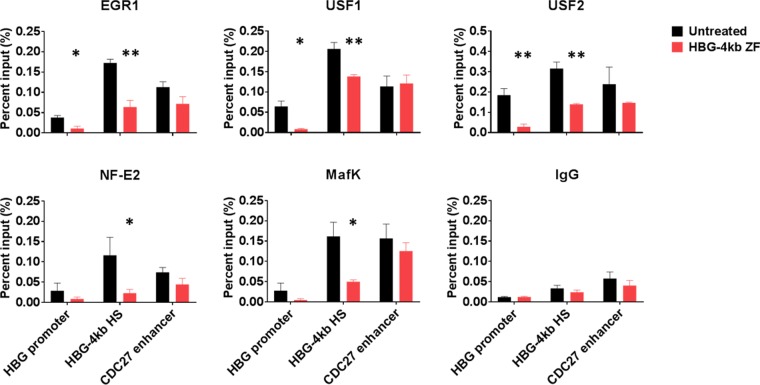
Reduced binding of transcription factors with the HBG-4kb HS in K562 cells expressing the HBG-4kb ZF. K562 cells (Untreated) or K562 cells exposed to 500 nM HBG-4kb ZF for 12 h were subjected to ChIP using antibodies specific for EGR1, USF1, USF2, NF-E2 (p45), MafK, or the negative control, IgG. The DNA was analyzed using primers specific for the γ-globin gene promoter (HBG promoter), the HBG-4kb HS (HGB-4kb), and a positive-control site, the CDC27 enhancer. The error bars reflect the SEM from three independent experiments (**, *P* < 0.01; *, *P* < 0.05).

To examine if the binding of the HBG-4kb ZF interfered with the binding of transcription factors at the HS at kb −4, we performed ChIP experiments in K562 cells 12 h after delivery of the ZF-DBD (Fig. 5). The binding sites for EGR1 and USF are located in close proximity (USF) or overlap (EGR1) the HBG-4kb ZF target site (Fig. 2A). We thus focused our attention on these proteins as well as on the NF-E2 and MafK transcription factors, as they have been shown to interact with USF2 (24). We examined the binding of these proteins to the HS at kb −4 as well as to the Gγ-globin gene promoter and a positive-control site, a regulatory element associated with the CDC27 gene. ENCODE data show that the CDC27^−^-associated regulatory DNA element associates with USF1, USF2, EGR1, NF-E2, and MafK, just like the HBG-4kb HS (Fig. S2). The binding of the transcription factors at the Gγ-globin gene promoter was relatively low, except for USF2, and delivery of the HBG-4kb ZF reduced the binding of the proteins to this site. Importantly, elevated binding of EGR1, USF1, USF2, NFE2, and MafK at the HBG-4kb HS was reduced after delivery of the HBG-4kb ZF to K562 cells. In contrast, there was no statistically significant reduction in the interaction of these proteins with the CDC27 enhancer after treatment with the HBG-4kb ZF. The data demonstrate that binding of the HBG-4kb ZF interfered with the binding of transcription factors at the HBG-4 kb HS.

**FIG 6:**
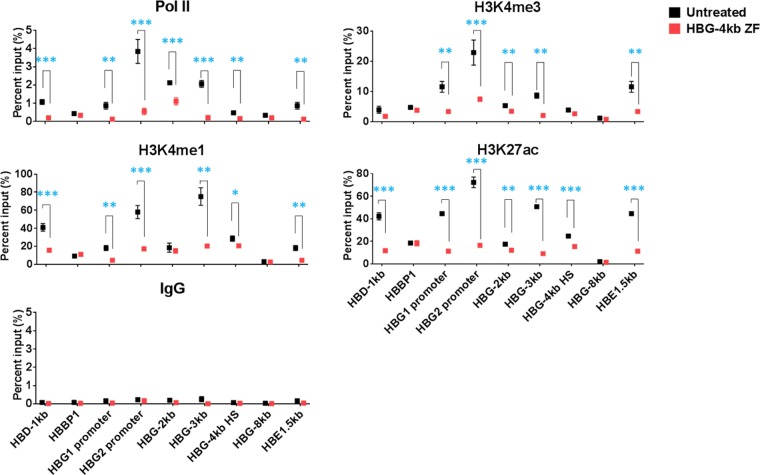
Altered histone modifications and Pol II interactions at the γ-globin promoter regions and at the HBG-4kb HS in K562 cells harboring the HBG-4kb ZF. K562 cells (Untreated) and K562 cells exposed to the HBG-4kb ZF were subjected to ChIP using antibodies specific for Pol II, H3K4me3, H3K4me1, H3K27ac, or IgG. The precipitated DNA was analyzed using primers specific for the HBD-1kb HS, the HBBP1 region (Fig. 1A), the Aγ-globin promoter (HBG1 promoter), the Gγ-globin promoter (HBG2 promoter), the HBG-2kb site, the HBG-3kb site, the HBG-4kb HS, the HBG-8kb site, and the HBE1.5kb site. The error bars reflect the SEM from three independent experiments (*, *P* < 0.05; **, *P* < 0.01; ***, *P* < 0.001).

We next analyzed Pol II binding and histone modification patterns at six different regions in the globin gene locus in K562 cells (WT) and in K562 cells treated with the HBG-4kb ZF (Fig. 6) using the ChIP assay. The regions that we analyzed were the HBG-4kb HS; a region located 4 kb upstream (HBG-8kb) or 1 and 2 kb downstream (HBG-3kb and HBG-2kb, respectively) of the HBG-4kb HS; the Gγ-globin (HBG2) and Aγ-globin (HBG1) gene promoter regions; a control region located downstream of the γ-globin genes (HBBP1); the HBD-1kb region, which has previously been shown to regulate accessibility at the HBG-4kb HS; and a region located downstream of the embryonic ε-globin gene (HBE1.5kb) (Fig. 1A) (19). There were no associations of the histone marks or Pol II with the HBG-8kb region, consistent with the ENCODE data. Pol II was also absent at the HBBP1 site. All the other regions were associated with elevated levels of Pol II or histone marks, except for H3K4me3, which was restricted to the HBG promoters, the HBG-2kb site, the HBG-3kb site, and the HBE1.5kb site. Exposure of K562 cells to the HBG-4kb ZF caused a reduction in H3K4me1 levels at the HBG-4kb HS, the HBG-3kb site, the HBD-1kb site, the HBE1.5kb site, and the two γ-globin promoters. The H3K27ac levels were reduced at the HBG-4kb HS, the HBG-3kb site, the HBG-2kb site, the HBD-1kb site, the HBE1.5kb site, as well as the Gγ- and Aγ-globin promoter regions in cells exposed to the ZF-DBD; the same was true for the binding of Pol II. The H3K4me3 levels were significantly reduced at the Gγ- and Aγ-globin promoter regions as well as at the HBE1.5kb site. There was also a small reduction at the HBG-2kb and the HBG-3kb sites after treatment with the ZF-DBD, which was statistically significant. We did not observe changes in histone modifications or Pol II binding at the HBBP1 site. The data demonstrate that the HBG-4kb HS plays an important role in setting up positive histone modifications within a 4-kb domain upstream of the Gγ-globin transcription start site, at a region located upstream of the adult δ-globin gene, and at a region located downstream of the embryonic ε-globin gene.

**FIG 7:**
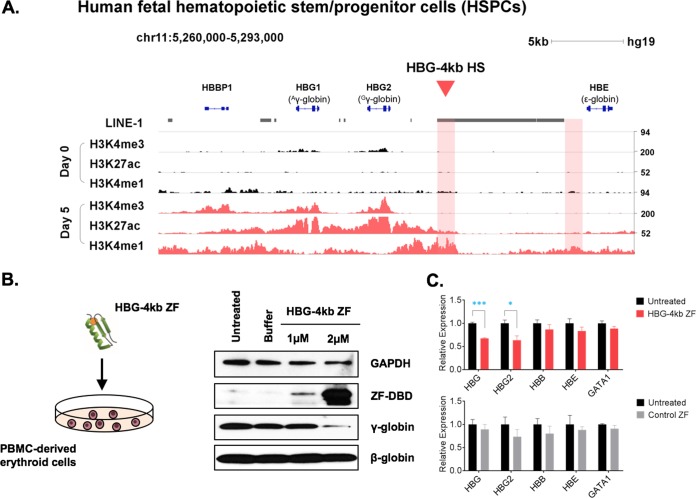
Reduced γ-globin gene expression in PBMC-derived human erythroblasts after delivery of the HBG-4kb ZF. (A) Histone modifications at the Gγ-globin upstream region in human fetal liver HPSCs before (day 0) and after (day 5) induction of differentiation (20). (B) Western blotting of the HBG-4kb ZF (ZF-DBD), GAPDH, γ-globin, and β-globin proteins in untreated differentiating PBMCs, in PBMCs treated with buffer, and in PBMCs treated with 1 μM HBG-4kb ZF for 24 h or with 2 μM HBG-4kb ZF for 48 h, with the ZF-containing medium being changed after 24 h. (C) Expression analysis of genes in erythroblasts in the absence or presence of the HBG-4kb ZF or the negative-control ZF-DBD (Control ZF). The ZF proteins were delivered to PBMC-derived erythroblasts for 12 h. RNA was extracted from erythroblasts incubated with the HBG-4kb ZF (top), with the control ZF (bottom), or without ZF-DBD (Untreated) and reverse transcribed. The cDNA was analyzed by qPCR using primers specific for the γ-globin (HBG), Gγ-globin (HBG2), adult β-globin (HBB), embryonic ε-globin (HBE), and GATA1 genes. Error bars reflect the SEM from three independent experiments (***, *P* < 0.001; *, *P* < 0.05).

To examine the function of the HBG-4kb HS in primary human erythroid cells, we analyzed previously published ChIP-seq data from differentiating primary human fetal liver hematopoietic stem and progenitor cells (HSPCs) (Fig. 7A) (20). The histone modification patterns of the γ-globin region were very similar to the pattern in K562 cells (Fig. 1A). Just like in the K562 cells, the active enhancer-associated histone marks H3K4me1 and H3K27ac were elevated at the HBG-4kb HS, and the elevated levels of these marks extended toward the Gγ-globin gene, further suggesting that this element functions as a chromatin opening and boundary element. To analyze the role of the HBG-4kb HS in primary human erythroid cells, we delivered the HBG-4kb ZF as well as the control ZF to differentiating peripheral blood mononuclear cells (PBMCs) (Fig. 7B and C). These primary erythroid cells derived from adult blood cells express high levels of adult β-globin and low levels of γ-globin (26). The γ-globin expression levels detected during the course of differentiation are shown in Fig. S3. Delivery of the HBG-4kb ZF to these cells caused a reduction in γ-globin protein without impairing expression of GAPDH or β-globin (Fig. 7B). Exposure of the primary cells to the HBG-4kb ZF reduced the mRNA expression of Gγ-globin and overall γ-globin gene expression (Fig. 7C). Expression of the adult β-globin, the embryonic ε-globin, and the GATA1 genes was not affected by the HBG-4kb ZF. Delivery of the control ZF did not cause any change in the expression of the γ-globin, β-globin, ε-globin, or GATA1 genes.

## DISCUSSION

The chicken, murine, and human β-globin gene loci have been the subject of intense study since the cloning of the genes in the late 1970s and early 1980s (33). Many fundamental chromatin and gene regulatory mechanisms were first described in the globin gene loci, including changes in the chromatin accessibility of the globin genes during erythroid differentiation (34), discovery of the first locus control region (35–37), chromatin architectural changes that bring genes and regulatory elements in close proximity (38, 39), the role of gene order (40, 41), and the discovery that enhancers recruit transcription complexes and initiate formation of noncoding eRNA (42–45). Despite this extensive body of work over a long time, new *cis*- and *trans*-regulatory activities that modulate the developmental stage-specific expression of the globin genes continue to be discovered (18, 19). Here we examined a hitherto uncharacterized DNase I HS located 4 kb upstream of the Gγ-globin gene. The results demonstrate that this element functions as an enhancer and suggest that active chromatin initiates within this element and spreads toward the Gγ-globin gene.

The fetal globin genes are regulated by positive and negative *cis*- and *trans*-regulatory activities that restrict expression of the genes to erythroid cells differentiating in the fetal liver. There are a number of positive regulatory elements located in proximal promoter or distal enhancer elements that mediate high-level expression of the γ-globin genes during the fetal period (15). Among those are the LCR, a 3′ enhancer, and DNA-binding sites for activating transcription factors in the promoter region. There are also a number of negative regulatory elements that repress γ-globin gene expression in adult erythroid cells, including repressor binding sites in the promoter and *trans*-acting repressor proteins like BCL11A (15–17). Mutations in promoter proximal repressor sites or in regulatory elements activating BCL11A expression cause HPFH (15–17). We demonstrate here that the HBG-4kb HS is a positive element for fetal globin gene expression.

In K562 cells, the HBG-4kb HS associates with a variety of transcription factors, including the ubiquitously expressed USF1, USF2, and EGR1 transcription factors as well as the tissue-restricted transcription factor NF-E2. We previously demonstrated that USF2 and NF-E2 together mediate the association of transcription complexes with the LCR and the adult β-globin gene promoter (24). Indeed, according to publicly available ChIP-seq and GRO-cap sequencing data, the HBG-4kb HS recruits Pol II and initiates transcription of noncoding RNA that primarily proceeds toward the γ-globin genes. Interestingly, the positive enhancer-associated histone marks H3K4me1 and H3K27ac are located at the HS at kb −4 and elevated levels extend toward the Gγ-globin gene promoter. We suggest that these histone marks are introduced through the action of proteins that associate with the site at kb −4 and that are perhaps propagated into the region between the enhancer and the promoter by the transcribing Pol II. The interaction of the HBG-4kb ZF with the HS at kb −4 reduced the interaction with Pol II at the enhancer and also reduced the elevated levels of H3K27ac at the enhancer and the promoter. The HBG-4kb ZF-mediated reduction of Pol II binding and transcription of the Gγ-globin gene could thus be a consequence of alterations in histone modifications and Pol II recruitment at the HBG-4kb HS.

Enhancers affect target gene expression in different ways (1). Most enhancers initiate the formation of noncoding eRNA, often in a bidirectional manner (8, 9). This is also true for the HSs associated with the LCR. Recent data suggest that eRNAs associate with CBP and that the function of eRNAs is to deliver CBP activity to promoter regions (46). Other data have shown that the process of noncoding transcription initiated at enhancers, rather than the transcripts, mediates chromatin accessibility and/or the relocation of genes into transcriptionally active regions of the nucleus (10, 47). We propose that the HBG-4kb HS functions by initiating unidirectional chromatin opening that may or may not be mediated by a transcription process. The region upstream of the HBG-4kb HS is devoid of the H3K4me1 and the H3K27ac marks. This is true for K562 and for primary fetal liver erythroid cells. This region contains two LINE-1 elements (L1PA7 and L1MA4A) which may cause low sequence reads. Nevertheless, the fact that transcription occurs mainly toward the γ-globin genes and the observation that the binding of the HBG-4kb ZF reduces histone marks throughout this region support the notion that the enhancer-mediated changes in chromatin structure proceed unidirectionally toward the genes.

**FIG 8:**
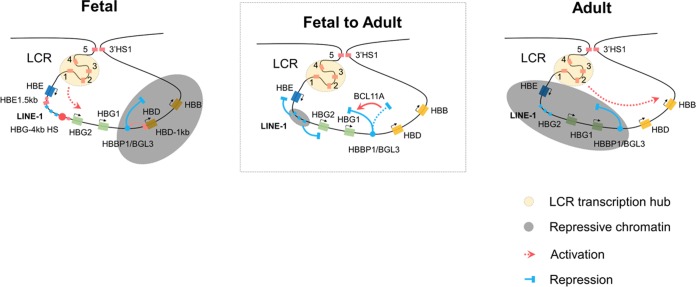
Model depicting the hypothetical role of the HBG-4kb HS, HBD upstream elements, and a LINE-1 element during fetal-to-adult β-globin gene switching. Repressive chromatin conformations are differentially formed over the embryonic, fetal, and adult globin genes during development. HBD upstream elements and the HBG-4kb HS regulate chromatin accessibility over the fetal and adult domains. A LINE-1 element located between the embryonic and fetal genes may be involved in the formation of repressive chromatin during the adult stage.

The HBG-4kb HS likely functions in the context of other DNA regulatory elements in the β-globin gene locus. For example, a previous study identified a chromatin-opening element located downstream of the murine embryonic β-type globin gene (HS-E1) (48). Histone modifications and Pol II binding at the corresponding human element (HBE1.5kb) are reduced in cells exposed to the HBG-4kb ZF, suggesting functional interactions between the two elements. Furthermore, a deletion of an HS located close to the adult δ-globin gene (HBD-1kb) reduced chromatin accessibility at the distal 3′HS1 site and the HBG-4kb HS (19). Likewise, we show here that interfering with the function of the HBG-4kb HS reduced the levels of active histone marks and Pol II at the HBD-1kb site. This suggests that these elements interact functionally to modulate globin gene expression during development. This is supported by Hi-C data suggesting interactions between these elements (19). However, the HBD-1kb site was shown to repress γ-globin expression, while the HBG-4kb HS functions as a positive element. The HBD-1kb site has been shown to interact with the PYR complex, a coregulator complex recruited by Ikaros (49). PYR contains proteins of the SWI/SNF chromatin remodeling complexes that positively regulate transcription as well as components of the NURD repressor and can thus function as activator and repressor (50). We propose that the HBG-4kb HS as well as elements downstream of the embryonic gene (HBE1.5kb) and upstream of the HBD gene, including HBBP1/BGL3 and HBD-1kb, are involved in regulating the developmental switch from fetal to adult globin gene expression (Fig. 8). At the fetal stage, the HBG-4 kb HS as well as the HBD upstream elements mediate an open chromatin structure of the γ-globin genes. At the adult stage, the HBD upstream elements contribute to the establishment of repressive chromatin containing the embryonic and fetal globin genes. LINE-1 elements located upstream of the HBG-4kb site and downstream of the adult β-globin genes could also be involved in regulating globin gene switching. The LINE-1 element between the ε- and γ-globin genes is conserved in primates closely related to humans (see Fig. S1 in the supplemental material). LINE-1 elements are usually associated with a repressive chromatin configuration, and during the adult stage the repressive chromatin may spread into the embryonic and fetal globin genes, while at the fetal stage the HBG-4kb HS and other regulatory DNA elements may prevent the spreading of heterochromatin toward the fetal globin genes.

## MATERIALS AND METHODS

### Bioinformatics analysis.

The raw Sequence Read Archive (SRA) data set of GRO-cap sequencing and ATAC-seq data for WT HBD-1kb and the HBD-1kb knockout (KO) were downloaded from GEO accession numbers GSM1480321, GSM2695562, as well as GSM2695565 and aligned to the human genome hg19 by the Bowtie 2 program with default settings (51). A bigwig file was generated using BAM to BigWig under the Galaxy project online tools (52). Snapshots of the genome features were visualized in the Integrative Genomics Viewer (IGV) (53), using hg19 as the reference genome. Data sets for RNA-seq, DNA-seq, Pol II, H3K4me1, H3K4me3, H3K27ac, EGR1, USF1, USF2, NF-E2, and MafK were extracted from the ENCODE database. The GRO-cap sequencing and ATAC-seq data were uploaded for the WT and HBD-1kb KO (54). The ChIP-seq data sets of histone modifications (H3K4me1, H3K4me3, and H3K27ac) in human fetal liver HPSCs (day 0 and day 5) were retrieved from GEO accession numbers GSM1816066, GSM1816067, GSM1816070, GSM1816071, GSM1816073, and GSM1816074 (20). The snapshot of the histone modifications at the Gγ-globin upstream region was captured within the Integrative Genomics Viewer (IGV). The human LINE data set was retrieved from the IGV server using the RepeatMasker tag of variations and repeats (55).

### ZF-DBD design and construction.

The HBG-4kb ZF and the negative-control ZF-DBD (control ZF) were designed using the Zinc Finger Tools website (http://www.scripps.edu/barbas/zfdesign/zfdesignhome.php) (56). The HBG-4kb ZF was designed to target 18 bp in the HBG-4kb HS (chr 11, positions 5279876 to 5280094; 5′-GTGGGGGAAAGCAAGCAG-3′). The amino acid sequences for the variable helical region of each zinc finger (ZF) module are as follows: F1, RADNLTE; F2, RKDNLKN; F3, ERSHLRE; F4, QSSNLVR; F5, RSDKLVR; and F6, RSDELVR. The control ZF was designed to target an 18-bp sequence located 158 bp upstream of the human γ-globin promoters (5′-AACGGTCCCTGGCTAAAC-3′). The amino acid sequences for the variable regions are as follows: F1, DSGNLRV; F2, QNSTLTE; F3, RSDHLTT; F4, SKKHLAE; F5, TSGHLVR; and F6, DSGNLRV. The ZF-DBDs harbored a 6×His tag and a 3×FLAG tag for purification and detection purposes, respectively (26, 29). The oligonucleotide and primer sequences used to construct the ZF-DBD-coding sequences are listed in Table S1 in the supplemental material.

### ZF-DBD expression and purification.

The HBG-4kb ZF and control ZF were expressed in and purified from E. coli as described previously with minor modifications (26, 29). BL21(DE3) (Sigma) competent cells harboring the ZF-DBD expression plasmids were incubated with 1 mmol/liter IPTG (isopropyl-β-d-1-thiogalactopyranoside; Sigma) at 37°C for 4 h. The cells were lysed using a French press with the high-power mode for 3 cycles. The purification of the ZF-DBDs was done with HisLink resin-packed columns (Promega, Madison, WI) as previously described (29). The supernatant of the cell lysate was passed through the resin column and washed with 5 column volumes of wash buffer (100 mmol/liter HEPES, 10 mmol/liter imidazole, pH 7.5) and eluted with 5 column volumes of elution buffer (100 mmol/liter HEPES, 1 mol/liter imidazole, pH 7.5). Desalting and concentration of the purified ZF-DBDs were done using 3-kDa-cutoff Vivaspin columns (GE Healthcare, Pittsburgh, PA) following the manufacturer's instructions. The concentration of purified ZF-DBDs was determined using bovine serum albumin (BSA) standards (NEB, Ipswich, MA).

### Generation of CRISPR/Cas9-mediated deletions within the HBG-4kb HS.

A guide RNA (gRNA) targeting the HBG-4kb HS was designed using the CRISPR DESIGN tool (http://crispr.mit.edu/). The corresponding oligonucleotide sequences were 5′-CACCGACCAACCTTTTGAGTTGCA-3′ for the forward sequence and 5′-AAACATGCAACTCAAAAGGTTGGTC-3′ for the reverse sequence. Ligation of the gRNA encoding the double-stranded oligonucleotide into the lentiCRISPRv2 vector (Addgene, Cambridge, MA) was performed as described by Sanjana et al. (32). The resulting vector, referred to as lentiCRISPRv2/HGB-4kb, was cotransfected together with the pVSVg (Addgene) and psPAX2 (Addgene) packaging plasmids into HEK293T cells for lentivirus production as previously described (32). After centrifugation, the supernatant containing the lentivirus particles was added to 1 × 10^6^ K562 cells. Transduced K562 cells were subjected to selection with 1 μg/ml puromycin (Sigma, St. Louis, MO). DNA from single-cell clones was extracted using a QIAamp DNA minikit (Qiagen, Hilden, Germany) and subjected to PCR using primers spanning the HBG-4kb HS (forward primer, 5′-ACACATCCTCACTGGGGAAC-3′; reverse primer, 5′-ATCAGCAGAGGCAGTCAGGT-3′). The PCR products were subjected to Sanger sequencing using the HBG-4kb sequencing primer (5′-GAGGGCCAAGCAAAATACAG-3′). K562 cells were subjected to karyotype analysis by the UF Pathology Core.

### Electrophoretic mobility shift assays.

The purified ZF-DBDs (1 to 100 nmol/liter) were incubated at room temperature for 30 min in a 20-μl reaction mixture mixed with 15 nmol/liter Cy5-labeled double-stranded DNA oligonucleotides, 100 ng herring sperm DNA, and binding buffer (10 mmol/liter Tris, pH 7.5, 50 mmol/liter KCl, 5 mmol/liter MgCl_2_, 100 μmol/liter ZnCl_2_, 1 mmol/liter dithiothreitol [DTT], 0.05% Triton X-100, 2.5% glycerol). A 10-μl volume of each reaction mixture was loaded onto 6% Tris-borate-EDTA gels (Invitrogen, Carlsbad, CA). The detection and analysis of the fluorescent signals as well as the determination of the dissociation constant (*K_d_*) were done as described previously (26, 29). The DNA oligonucleotide sequences used in the EMSAs are listed in Table S2.

### Cell culture and stable transfection.

K562 cells were grown in RPMI medium (Cellgro, Manassas, VA) supplemented with 10% (vol/vol) fetal bovine serum (FBS; Sigma) and 1% (vol/vol) penicillin-streptomycin (P-S; Cellgro) in the presence of 5% CO_2_ at 37°C. The cell density was maintained at 10^6^ cells/ml. K562 cells were transfected with the pMSCVneo vector containing the HBG-4kb ZF using the Lipofectamine 2000 reagent (Invitrogen). Transfected cells were maintained under Geneticin (Sigma) selection. Human peripheral blood mononuclear cells (PBMCs; StemCell Technologies, Vancouver, Canada) were expanded and differentiated into erythroblast cells following the supplier's instructions. The PBMCs (15 × 10^6^ cells) were cultured in StemSpan SFEM II medium (StemCell Technologies), StemSpan erythroid expansion supplement, and 1% (vol/vol) P-S. The cells were incubated in the presence of 5% CO_2_ at 37°C, and the medium was changed every 2 days.

### Direct delivery of ZF-DBD proteins into K562 cells and PBMCs.

Delivery of ZF-DBDs into K562 cells and PBMCs was performed using a direct protein delivery protocol (26, 29). Cells (2 × 10^6^ cells/ml) were supplied with purified ZF-DBD proteins (from 500 nmol/liter to 2 μmol/liter) for various times. Cells were collected and subjected to Western blotting, chromatin immunoprecipitation (ChIP), and reverse transcription (RT) followed by quantitative PCR (qPCR).

### Western blotting.

Western blotting procedures were performed using a previously published protocol (26). Briefly, cells were collected and lysed in radioimmunoprecipitation assay (RIPA) buffer (10 mmol/liter Tris-HCl, pH 8.0, 1 mmol/liter EDTA, 0.5 mmol/liter EGTA, 1% Triton X-100, 0.1% sodium deoxycholate, 0.1% SDS, 140 mmol/liter NaCl, 1× protease inhibitor). Cytoplasmic and nuclear proteins were separated by applying NE-PER nuclear and cytoplasmic extraction reagents (Pierce, Rockford, IL) according to the manufacturer's instructions. The supernatant containing the proteins was loaded onto 4 to 15% (wt/vol) SDS-PAGE gels (Bio-Rad, Hercules, CA). After electrophoresis the proteins were transferred to polyvinylidene difluoride (PVDF) membranes (Bio-Rad). The PVDF membranes were incubated with anti-FLAG (catalog number F3165; Sigma), anti-GAPDH (catalog number CST5174; Cell Signaling, Beverly, MA), anti-CTCF (catalog number 2899; Cell Signaling), anti-γ-globin (catalog number sc-21756; Santa Cruz Biotechnology, Santa Cruz, CA), anti-GATA1 (catalog number sc-265; Santa Cruz Biotechnology, Santa Cruz, CA), or anti-Brg1 (catalog number sc374197; Santa Cruz Biotechnology, Santa Cruz, CA) antibodies. The ZF antiserum was provided by the laboratory of Carlos Barbas III (Scripps Research Institute, CA). The membranes were washed and incubated with secondary anti-rabbit (catalog number sc-2004; Santa Cruz Biotechnology, Santa Cruz, CA) or anti-mouse (catalog number sc-2005; Santa Cruz Biotechnology, Santa Cruz, CA) antibodies. Antibodies were detected using an enhanced chemiluminescence (ECL) reagent (Millipore, Danvers, MA) and visualized with X-ray films (Kodak, Rochester, NY).

### ChIP.

The chromatin immunoprecipitation (ChIP) protocol used in this study was described previously (27). Briefly, 5 × 10^6^ cells were collected and cross-linked with 1% (vol/vol) formaldehyde at room temperature for 10 min. The reaction was quenched with 125 mmol/liter glycine, and the cells were incubated in cell lysis buffer (5 mmol/liter PIPES [1,4-piperazinediethanesulfonic acid], pH 8.0, 85 mmol/liter KCl, 0.5% NP-40 [octylphenoxypolyethoxylethanol], 1× protease inhibitor) and nucleus lysis buffer (50 mmol/liter Tris, pH 8.0, 10 mmol/liter EDTA, 0.32% SDS, 1× protease inhibitor). Chromatin was fragmented to average 300-bp fragments on ice using a Bioruptor disruptor (Diagenode, Denville, NJ). For each immunoprecipitation reaction, chromatin from 2 × 10^5^ cells was incubated with 2.5 μg antibody plus protein A/G magnetic beads (Pierce) rotating at 4°C overnight. The chromatin-antibody-bead complexes were subjected to washing, eluting, and reverse cross-linking at 68°C overnight as described previously (27). The DNA was purified using a PCR purification kit (Qiagen) and subjected to qPCR as described earlier. The antibodies used in the ChIP and Western blotting experiments are listed in Table S3. The sequences of the primers used in the ChIP assays are listed in Table S4.

### RNA extraction and gene expression analysis.

Total RNA was extracted using an RNeasy kit (Qiagen, Hilden, Germany) following the manufacturer's protocol. The RNA was reverse transcribed into cDNA with an IScript cDNA synthesis kit (Bio-Rad). The cDNAs were subjected to qPCR in a 10-μl reaction mix with SYBR green and 1.5 μmol/liter of each forward and reverse primer. Gene expression was analyzed by the normalization of expression to that of GAPDH using the Δ*C_T_* method. The primer sequences used for RT-qPCR are listed in Table S5.

### Statistics.

The experiments were repeated at least three times unless stated otherwise. In experiments involving qPCR, each sample from the three repeats was analyzed by PCR three times. Error bars represent the standard error of the mean (SEM). The statistical analysis was performed with GraphPad Prism software.

## Supplementary Material

Supplemental file 1
