# Supplementary material for: Identification of a Novel Enhancer/Chromatin Opening Element Associated with High-Level γ-Globin Gene Expression
Source: Mol Cell Biol. 2018 Sep 14;38(19):e00197-18. doi: 10.1128/MCB.00197-18 (PMC6146835; doi:10.1128/MCB.00197-18)
Supplement: Supplemental file 1 [file zmb999101836s1.pdf]

Supplementary Table 1

ZF-DBD construct oligunucleotides and primers	
HBG-4kb ZF_v1	GCCCGTGTGGGTACGCTGATGCTCGGTCAGGTTGTCGGCCCGCGAGAACGACTTGCCACA
HBG-4kb ZF_v2	CCTGTATGCGTCCGTTGATGGTTCTTGAGATTATCCTTTCGGCTGAAGGATTTACCGCAT
HBG-4kb ZF_v3	GTGTGAGTGCGCTGGTGCTCCCGCAGGTGGGACCGCTCACTAAAACTCTTCCCACATTCG
HBG-4kb ZF_v4	GCCCGTGTGGGTACGCTGATGCCGCACCAGGTTGGAGGACTGCGAGAACGACTTGCCACA
HBG-4kb ZF_v5	CCTGTATGCGTCCGTTGATGTCGCACGAGCTTGTCAGACCTGCTGAAGGATTTACCGCAT
HBG-4kb ZF_v6	CGGTGTGAGTGCGCTGGTGACGAACCAGTTCGTCTGACCGACTAAAACTCTTCCCACATT
HBG-4kb ZF_C1	CGGGGAGAAACCCTATAAGTGTCCGGAGTGTGGCAAGTCGTTCTC
HBG-4kb ZF_C2	GCGTACCCACACGGGCGAAAAGCCGTACAAATGCCCAGAATGCGGTAAATCCTTCAGC
HBG-4kb ZF_C3	TCAACGGACGCATACAGGAGAGAAGCCATACAAATGTCCCGAATGTGGGAAGAGTTTTAG
HBG-4kb ZF_C4	TATAAGTGTCCGGAGTGTGGCAAGTCGTTCTC
HBG-4kb ZF_F(1-3)	CAGGACGAATTCATGCCGAAAAAAAAACGCAAAGTGCTAGAGCCCGGGGAGAAACCCTATAAG
HBG-4kb ZF_R(1-3)	ATGACCGGTGTGAGTGCGCTGGTG
HBG-4kb ZF_F(4-6)	GACACCGGTGAGAAGCCCTATAAGTGTCCGGAGTGTGG
HBG-4kb ZF_R(4-6)	GACAGGCCTTCATTTATCATCATCATCTTTATAATCTTTATCATCATCATCTTTATAATCTTTATCA TCATCATCTTTATAATCGCTGGTTTTTTTGCCGGTGTGAGTGCGCTGGTG
Control ZF_v1	GCCCGTGTGGGTACGCTGATGGACTCTTAGATTGCCTGAGTCCGAGAACGACTTGCCACAC
Control ZF_v2	CCTGTATGCGTCCGTTGATGCTCCGTAAGAGTCGAGTTCTGGCTGAAGGATTTACCGCAT
Control ZF_v3	TTGTGAGTGCGCTGGTGCGTCGTTAAGTGATCAGATCGACTAAAACTCTTCCCACATTCG
Control ZF_v4	GCCCGTGTGGGTACGCTGATGCTCTGCAAGATGCTTCTTCGACGAGAACGACTTGCCACAC
Control ZF_v5	CCTGTATGCGTCCGTTGATGTCTGACTAGATGTCCACTAGTGCTGAAGGATTTACCGCAT
Control ZF_v6	CGGTGTGAGTGCGCTGGTGGACTCTTAGGTTACCTGAGTCACTAAAACTCTTCCCACATTCG

Supplementary Table 2

EMSA oligos	
HBG-4kb_on-target_F	GAGAACTTAGTGGGGGAAAGCAAGCAGAGAGTATAT
HBG-4kb_on-target_R	ATATACTCTCTGCTTGCTTTCCCCCACTAAGTTCTC
HBG-4kb_off-target_F	GAGAACTTAGTGGGGGAAAAAAAGCAGAGAGTATAT
HBG-4kb_off-target_R	ATATACTCTCTGCTTTTTTTCCCCCACTAAGTTCTC

Supplementary Table 3

ChIP antibodies	
rabbit IgG	Santa Cruz Biotechnology (sc2027)
αRNA Pol II	Abcam (ab8408)
αH3K4me3	Abcam (ab8580)

αH3K27ac	Abcam (ab4729)
αH3K4me1	Abcam (ab8895)
αEGR1	Cell Signaling (15F7)
αUSF1	Santa Cruz Biotechnology (sc8983X)
αUSF2	Santa Cruz Biotechnology (sc861X)
αNF-E2	Santa Cruz Biotechnology (sc477)
αMafK	Santa Cruz Biotechnology (sc291X)
αFLAG	Sigma (F3165)
**Western Blotting antibodies**	
ZF-DBD	Carlos Barbas lab, Scripps Research Institute, California
γ-globin	Santa Cruz Biotechnology (sc21756)
GATA1	Santa Cruz Biotechnology (sc265)
BRG1	Santa Cruz Biotechnology (sc374197)
GAPDH	Novus Biologicals (NB300-327)
CTCF	Cell Signaling Technology (D31H2)
**Western Blotting secondary antibodies**	
Goat anti-rabbit IgG-HRP	Santa Cruz Biotechnology (sc-2004)
Goat anti-mouse IgG-HRP	Santa Cruz Biotechnology (sc-2005)

Supplementary Table 4

**ChIP qPCR primers**	
CDC27enhancer_F	AGAGCTGCTGGTCCTCCTAA
CDC27enhancer_R	TCCTTGAATACAGATTCCTCAGTG
HBG_Promoter_F	CTCAATGCAAATATCTGTCTG
HBG_ Promoter_R	TCTGGACTAGGAGCTTATTG
HBG1 promoter F	TTACTGCGCTGAAACTGTGG
HBG1 promoter R	TCAGTTAGCAGTGGTTTCTAAGG
HBG2 promoter F	GCTGAGATGAAACAGGCGTG
HBG2 promoter R	TGATGGGACACGTCTTAGTCTC
HBG-4kb ZF_F	GCTCACTGACTGCATGGAAA
HBG-4kb ZF_R	ACAATTATGGCTGCCTCTGC
HBG-4kb HS_F	ACACATCCTCACTGGGGAAC
HBG-4kb HS_R	ATCAGCAGAGGCAGTCAGGT
HBG-4kbHistoneF	CTGGCAGGGAAGTAGGACAG
HBG-4kbHistoneR	CCCATGCAACTCAAAAGGTT
HBD-1kb_F	CCAGGAATGAAGATCCCAGT

HBD-1kb_R	TCTCTTCCCCTCTTCCTTCC
HBG-2kb_F	CTCAGCCTCCCAAGTAGCTG
HBG-2kb_R	CACGCCTGTAATCCCAGAAT
HBG-3kb_F	AAAAGCCCTTATTGCCTGTG
HBG-3kb_R	TCAAGGACGTGATGTTGAGG
HBG-8kb_F	AGACAGTGTGGCGATTCCTC
HBG-8kb_R	CATTTGGGTTGGTTCCAAGT
HBE1.5kb_F	GTGCATGGTTTCCTCCTGTT
HBE1.5kb_R	CCCAAGAGGAGCAGTGAGTC
3HS1_F	TTCAGGCCTTGCTCAACTCT
3HS1_F	TCTAGGCTTGGCTCCTGTTT
HBB_F	GTCAGGGCAGAGCCATCTAT
HBB_R	AACGGCAGACTTCTCCTCAG
HBD_F	TGCAGAGGAGAACAGGGTTT
HBD_R	CTGCCTTTTATGCTGGTCCT
HBBP1_F	TTTCTGCCTTTCTGCACCTT
HBBP1_R	TCACCAGGAAACTCCCAGAT
HBG_F	AGCCTTGTCCTCCTCTGTGA
HBG_R	TGACAAGGCAAACTTGACCA
HBE_F	GTCCATCCATCACTGCTGAC
HBE_R	GGCCTGAGAGCTTGCTAGTG
HS1_F	CACAACCGCAAGCTTATTGA
HS1_R	CCCCTAAGCTCCCAGAAAAC
HS2_F	CCACACAGGTGAACCCTTTT
HS2_R	GGACACATGCTCACATACGG
HS3_F	TGAGGGTCTTGTGTTTGCTG
HS3_R	GCAGTGCCAGACCTATGTGA
HS4_F	AGCTGCTGAGTGGGAGAGAG
HS4_R	GGCATCTAGCGCAATGACTT
Necdin_F	GTGTTATGTGCGTGCAAACC
Necdin_R	CTCTTCCCGGGTTTCTTCTC

Supplementary Table 5

RTqPCR primers	
GAPDH_F	GAAGGTGAAGGTCGGAGTCA
GAPDH_R	TTGAGGTCAATGAAGGGGTC
HBG_F	TGGATGATCTCAAGGGCAC
HBG_R	TTGCAGAATAAAGCCTATCCTTGA
HBG2_F	GATGCCATAAAGCACCTGGATG
HBG2_R	TTGCAGAATAAAGCCTATCCTTGA
HBB_F	GCACGTGGATCCTGAGAACT
HBB_R	GCCACCACTTTCTGATAGGC
HBE_F	GGGCTTGAGGTTGTCCATGTTT
HBE_R	CAATCACTAGCAAGCTCTCAGG
GATA1_F	CCACTACCTATGCAACGCCT
GATA1_R	ACCTGCCCGTTTACTGACAA

Supplementary Table 6

Analytical Comparison of ATAC-seq Signals in β-globin Locus Regions for HBD-1kb KO relative to WT

	3'HS1	HBB	HBD	HBG1	HBG2	HBG-4kb	HBE	HS1	HS2	HS3	HS4	HS5
WT	37.087	11.126	33.378	44.504	48.213	33.378	22.252	66.756	33.378	14.835	29.669	18.543
HBD-1kb KO	3.075	3.075	3.075	21.525	27.675	3.075	15.375	24.600	27.675	30.750	18.450	9.225
KO/WT Difference	-34.012	-8.051	-30.303	-22.979	-20.538	-30.303	-6.877	-42.156	-5.703	15.915	-11.219	-9.318
KO/WT Ratio	0.081	0.276	0.092	0.484	0.574	0.092	0.691	0.369	0.829	2.073	0.623	0.487
KO/WT Fold Change	-12.061	-3.618	-10.855	-2.068	-1.742	-10.855	-1.447	-2.714	-1.206	2.073	-1.608	-2.010

* KO/WT Difference as KO-WT; KO/WT Ratio as KO/WT; KO/WT Fold Change as (KO-WT)/KO

Supplementary Figure S1

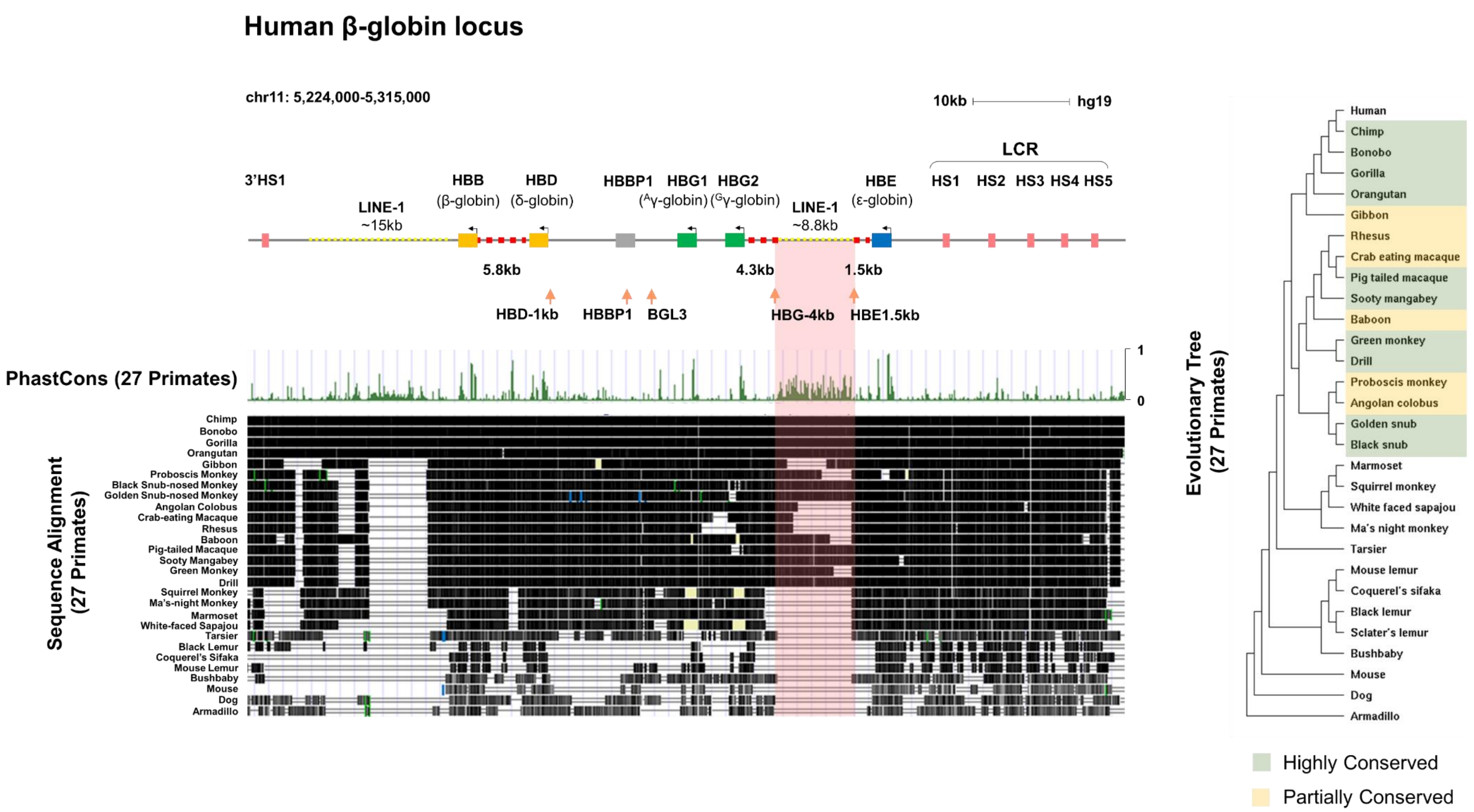

Figure S1: Localization and evolutionary conservation of LINE-1 elements in the human β-globin gene locus.

Supplementary Figure S2

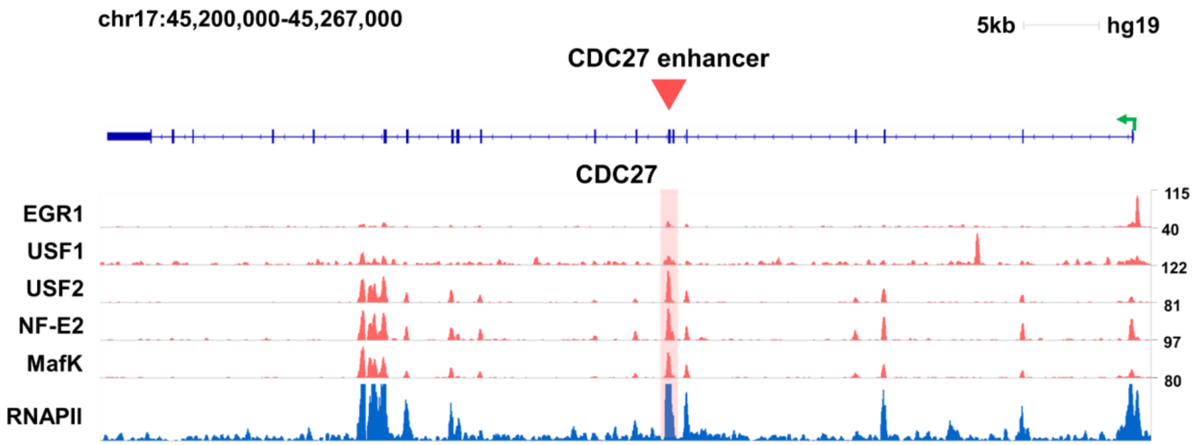

Figure S2: ChIP-seq. data for EGR1, USF1, USF2, NF-E2, MafK, and RNA polymerase II (RNAPII) at the CDC27 enhancer in K562 cells. Data were retrieved from the ENCODE project and GEO database.

Supplementary Figure S3.

A.

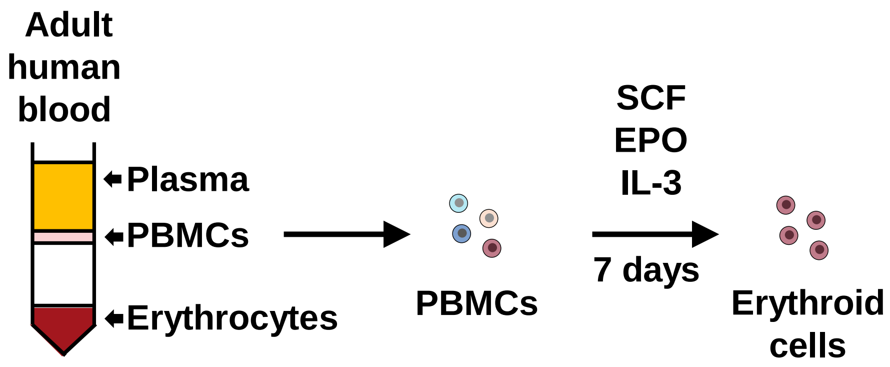

B.

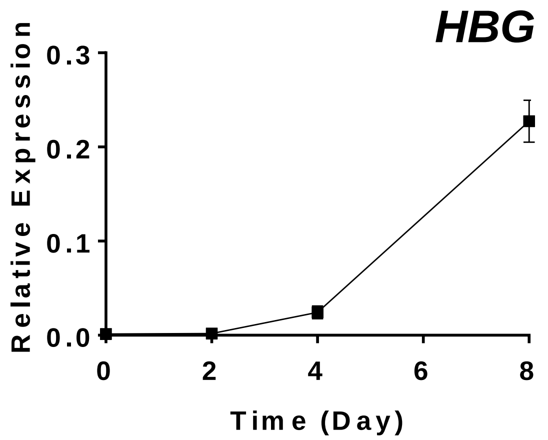

Figure S3: A. Experimental outline for the generation of erythroid cells from peripheral blood mononuclear cells (PBMCs). B. γ-globin expression profile during the 8 days of culture. RNA was extracted at the indicated time points and subjected to qPCR using primers specific for γ-globin. Error bars reflect the SEM from three independent experiments.
